# Under- and Normal-Weight Patients Are More Susceptible to Recurrence of Phyllodes Tumor

**DOI:** 10.1155/2022/4474251

**Published:** 2022-01-31

**Authors:** Yong Yeup Kim, Hayeon Kim, Woo Young Kim, Jai Hyun Chung, Jae Bok Lee, Sang Uk Woo

**Affiliations:** ^1^Department of Breast and Endocrine Surgery, Korea University Guro Hospital, Seoul, Republic of Korea; ^2^Department of Pathology, Korea University Guro Hospital, Seoul, Republic of Korea

## Abstract

**Purpose:**

Phyllodes tumors (PTs) of the breast are rare fibroepithelial neoplasms, and factors associated with the recurrence of PTs are poorly understood. This study sought to identify clinicopathological factors associated with the recurrence of PTs.

**Method:**

From January 2009 to December 2019, we identified 100 patients who underwent definitive surgery for PT. Clinicopathological risk factors associated with the recurrence of PT were assessed.

**Results:**

The median age of the patients was 44 y (range, 19–62 y), and the median tumor size was 4 cm (0.8–30 cm). At a median follow-up of 26.7 mo (0–103 mo), 22 of the 100 patients experienced local recurrence. In the univariate and multivariate analyses, body mass index ≥ 23 kg/m^2^ (*P* = 0.042 in the univariate analysis; *P* = 0.039 in the multivariate analysis), tumor size ≥ 5 cm (*P* = 0.006 in the univariate analysis; *P* = 0.036 in the multivariate analysis), and the presence of stromal overgrowth (*P* = 0.032 in the univariate analysis; *P* = 0.040 in the multivariate analysis) were associated with an increased risk of local recurrence. Resection margins and grade were not associated with local recurrence.

**Conclusion:**

Normal- or underweight patients and those with larger tumor sizes were more prone to local recurrence. Further larger, multicenter studies with a long-term follow-up are required.

## 1. Introduction

Phyllodes tumors (PTs) are rare breast fibroepithelial neoplasms that account for 0.3%–1% of all breast tumors [1, 2]. PTs are characterized by biphasic proliferation of both epithelial and stromal components [3]. However, in Asia, its prevalence has increased to 8% [4]. In general, PTs are classified as benign, borderline, or malignant according to the World Health Organization (WHO) classification and are based on a combination of several histological features, including stromal cellularity, nuclear atypia, mitotic activity, stromal overgrowth, and tumor margin appearance [5]. However, there are no definite criteria or clear cutoffs for the histological parameters. Moreover, diagnosis of PTs and distinction from fibroadenoma after core needle biopsy are challenging and sometimes ambiguous. Unlike fibroadenoma, PTs have the potential for recurrence, with the risk of local recurrence (LR) ranging from 17% in benign PTs to 27% in malignant PTs. Distant metastasis occurs in up to 22% of malignant PTs [5].

The National Comprehensive Cancer Network guidelines recommend wide excision with a margin of 1 cm or more for each definitive surgery for the treatment of PTs, regardless of the pathological grade [1]. Several retrospective studies have supported this guideline [6]. However, the recent study showed contradictory results. Laura et al. reported that a wider margin width was not associated with a decreased risk of LR, and even a positive margin did not impact LR for benign PTs [7].

Therefore, we looked for unknown clinicopathological risk factors associated with higher LR of PTs.

## 2. Materials and Methods

We reviewed the medical records at Korea University Guro Hospital after institutional review board of Korea University Guro Hospital approval (IRB no. 2021GR0285) based on ethical standards of the Declaration of Helsinki for patients with PTs. Between January 2009 and December 2019, all women diagnosed with PT after wide excision or total mastectomy for definitive surgical management were included in the study. The requirement for written informed consent was waived based on the retrospective design of the study. Patient demographic characteristics, clinicopathological data, and survival outcomes were recorded. All available hematoxylin and eosin-stained slides (of selected cases) were reviewed by a pathologist (HK). For the remaining cases, tumor characterization was based on the original pathology report.

Surgical decisions were made at the discretion of the breast surgeon based on the clinically estimated tumor size, breast size, the likelihood of obtaining negative margins, cosmesis, and patient preference. Histological grading was performed by trained breast pathologists based on the 1981 WHO guidelines.

PTs were classified as malignant if the following features were present: marked stromal cellularity and overgrowth, increased nuclear atypia, infiltrative tumor borders, and high mitotic activity of ≥10 mitoses per 10 high-power fields. Any PTs with heterologous sarcomatous elements were also deemed malignant. Borderline PTs were defined as the presence of some, but not all, malignant features. Tumor size was measured as the maximum diameter in the pathological evaluation. For tumors with multiple foci in the same breast, the size was recorded as the maximum diameter of the separate tumor. Multifocality and multicentricity were not distinguishable. The resection margin was defined as positive if tumors were present “on ink” and negative if absent. It included the superficial and deep margins. If reexcision was performed, the margin was based on the last surgical procedure. If the resection margin was recorded as negative, the measured width of the closest negative margin was recorded.

Recurrence-free survival (RFS) was defined as the time interval between initial diagnosis and the first detection of recurrence. LR was defined as pathologically proven tumor recurrence in a previously treated breast.

### 2.1. Statistics

The base of follow-up was defined as the detection of a recurrent tumor. Surgery due to reexcision was not considered during the follow-up. Optimal cutoff values for body mass index (BMI) were classified based on a BMI of 23 kg/m^2^ as the WHO Asian criteria-based BMI. The mass size was classified based on a tumor size of 5 cm. Stromal cellularity, stromal atypia, and mitosis were classified as a three-tiered grading system for PTs based on the 2012 WHO classification [5].

Continuous variables were summarized using median (range), and categorical variables were summarized using number (frequency). Comparisons between benign, borderline, and malignant histologic groups were carried out using the Kruskal–Wallis test for continuous variables and the Mantel–Haenszel test for categorical variables. The RFS rates were calculated using the Kaplan–Meier method. The cumulative incidence of LR was estimated using one minus the Kaplan–Meier estimate of local recurrence-free survival. Cox regression was used to calculate hazard ratios (HRs) to estimate the risk of locoregional recurrence by individual factors. Factors with *P* < 0.10 were entered for multivariable competing risks regression based on the Fine and Gray model. Adjusted subdistribution HRs and 95% confidence intervals (CIs) were calculated. Statistical significance for two-tailed *P* values was set at *P* < 0.05.

All statistical analyses were performed using IBM SPSS version 20 and the open-source statistical software JAMOVI version 1.6.23 (The jamovi Project, Sydney, Australia).

## 3. Results

We identified 100 women with PTs between January 2009 and December 2019. The median patient age was 44 y (IQR, 39–49 y) with a range of 19–62 y. The median pathological tumor size was 4 cm (IQR, 2.55–6.5 cm) with a range of 0.8–30 cm. The median patient BMI was 23.7 kg/m^2^ (IQR, 21.1–26.3 kg/m^2^) with a range of 16.1–31.9 kg/m^2^. PTs were classified as benign in 65% (*n* = 65), borderline in 29% (*n* = 29), and malignant in 6% (*n* = 6) of the cases (Table 1).

Overall, 92% of the patients (*n* = 92) underwent wide excision, and 8% underwent mastectomy (*n* = 8). None of the patients underwent lymph node evaluation. At the initial surgical procedure, 68% had a negative margin (*n* = 68) and 31% had a positive margin (*n* = 31). Two patients had lung metastasis, of whom one patient had a malignant phyllodes tumor and the other patient had a borderline phyllodes tumor. The borderline phyllodes tumor patient underwent video-assisted thoracic surgery left lower lobe lobectomy and received an adjuvant chemotherapy at the other hospital. The malignant phyllodes tumor patient died because of multiple lung metastases.

At a median follow-up of 26.7 mo (range, 0–103 mo), 22% (*n* = 22) of the patients experienced an LR, and none of the patients had a regional recurrence or distant recurrence. LR by grade occurred in 20.0% (*n* = 13) of 65 benign PTs, 24.1% (*n* = 7) of 29 borderline PTs, and 33.3% (*n* = 2) of six malignant PTs. A histological upgrade occurred in 22.7% (*n* = 5) of LRs (benign to borderline in four cases and benign to malignant in one case). Three patients showed histological downgrades in 13.6% (*n* = 3) (borderline to benign in three cases). LR occurred at a median of 22.5 mo, and 21 of the 22 LRs developed within 5 y, with the longest LR occurring over 8 y after surgery (range, 6–98 mo). All patients with recurrence underwent wide excision, except one patient (of the seven patients who experienced more than two recurrences) who underwent mastectomy. The final margin status of these 22 patients with an LR included 14 patients with a final negative margin, five patients with a final positive margin (one benign, three borderline, and one malignant), and three patients with unknown margins (two benign and one borderline).

In the univariate analysis, factors associated with a higher incidence of LR included a BMI ≥ 23 kg/m^2^ (HR = 0.405; 95% CI, 0.169–0.968; *P* = 0.042), pathological tumor size ≥ 5 cm (HR = 3.506; 95% CI, 1.423–8.637; *P* = 0.006), and aggravated stromal overgrowth (HR = 2.654; 95% CI, 1.086–6.484; *P* = 0.032). There was no significant association between LR and type of surgery, pathological grade, stromal hypercellularity, cellular atypia, mitosis, tumor border, and resection margin.

In the multivariate analysis, preoperative BMI (<23 kg/m^2^ vs. ≥23 kg/m^2^: HR = 0.386; 95% CI, 0.156–0.954, *P* = 0.039), pathological tumor size (<5 cm vs. ≥5 cm: HR = 2.671; 95% CI, 1.066–6.694, *P* = 0.036), and stromal overgrowth (absent vs. present: HR = 2.706; 95% CI, 1.046–6.999, *P* = 0.040) were associated with LR (Table 2).

Only two patients received adjuvant chemotherapy, and one patient received adjuvant radiotherapy; thus, the influence of these therapies on LR could not be analyzed. Almost all patients survived without disease, except for one patient who died of the disease.

## 4. Discussion

**Figure 1:**
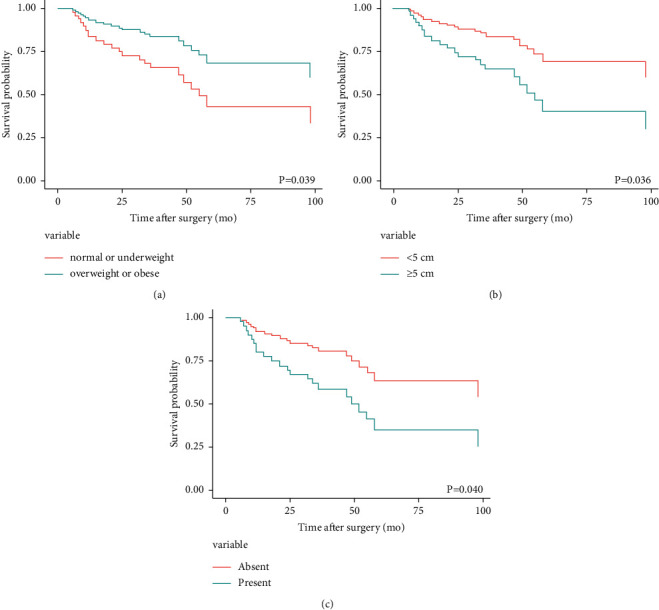
Adjusted local recurrence-free survival curve by a multivariate Cox proportional hazard model of phyllodes tumor. It is subdivided by the body mass index (a), tumor size (b), and stromal overgrowth (c).

This study aimed to predict the patient group that was expected to have a high recurrence rate by analyzing the factors affecting the recurrence of PTs and to improve the outcome through early detection of recurrence by performing close postoperative follow-up. In the results, a tumor size of ≥5 cm and a BMI of <23 kg/m^2^ were confirmed as factors that caused more recurrences after local excision of PTs, Figure 1.

This study enrolled 10 year-data and used appropriate statistical methods. So, the cohort is sufficient, and study results are suitable. Unfortunately, however, the median follow-up is close to the median disease-free survival. Therefore, long-term follow-up after surgery in patients with a tumor size ≥ 5 cm or underweight or normal weight according to the Asian standard BMI can reduce missed recurrences and increase patient satisfaction after surgery. With early detection, surgery can be performed with a minimal resection area.

PTs are, in many cases, indistinguishable from fibroadenoma because of the inaccuracy of core needle biopsy results and heterogeneity of PTs; vacuum-assisted breast excision (VAE) or surgical excision is chosen based on the clinical features. In general, when a fibroepithelial lesion appears on a core needle biopsy, surgical excision is recommended with the possibility of a PT in mind when the size is large or when it grows rapidly. By applying the results of this study, surgical excision rather than VAE may be recommended for patients with a tumor size of ≥5 cm or a BMI of <23 kg/m^2^, who are expected to have high risk of recurrence before the pathological diagnosis is confirmed. It is thought that it can be helpful to increase disease-free survival or decrease the recurrence rate; however, supporting data through additional research should be confirmed.

In our data, recurrence was common when the size was ≥5 cm or the BMI was <23 kg/m^2^, and the margin status was not related to recurrence. In a recent study, breast volume was found to be related to BMI [8]. That is, the larger the tumor size and the lower the BMI, the smaller the normal breast volume and the more likely the recurrence of PTs.

Many studies have reported that positive margins increase recurrence between surgical margins and recurrence [9–17]. However, based on recent studies, negative margins are not essential for lowering recurrence [18, 19], especially in the setting of a benign PT [20, 21].

Stromal overgrowth influences recurrence; however, the number of mitoses, borders, stromal atypia, and stromal cellularity, which affects the grade based on the WHO classification, did not affect recurrence in this study. Access to cellular atypia and stromal cellularity may be affected by the variability among pathologists. The evaluation of mitoses may also be affected by the number of fields to be analyzed and whether it should be a maximal or average mitotic count [14]. In our pathological report, stromal overgrowth was recorded as binomial; however, cellular atypia and stromal cellularity were expressed as multinomial. Therefore, the relationship between pathological factors and recurrence will be clear only when the clarity of pathological ambiguity is established. Until now, only histological features have been used in diagnosing PT; however, gene assays or immunohistochemistry are being studied for diagnosing and grading PT [22]. These studies are expected to clarify the distinction between PT and fibroadenoma and between the grades of PT.

In this study, reexcision was not performed in most patients, even if the margin was positive, and the margin status did not have a statistical significance in recurrence. Nevertheless, the recurrence rate was not significantly different from the recurrence rate reported in a previous study [5], which was generally 20%. These results suggest that the benefit of reexcision is not significant when R1 resection is performed. However, the superficial and deep margins were not distinguished from the margins, and the total number of patients was insufficient.

The change in the histological grade upon LR is unusual, and our study showed only one case of malignant transformation. Although most of the locally recurrent tumors tended to follow their primary form, the histological transformation was seen not only in upgrading but also in downgrading. Considering the histopathological diversity of PT and the ambiguous criteria for histological grading, histological downgrading due to insufficient sampling or discrepancies in pathological evaluation cannot be excluded. Additional investigations are required to identify the clinical significance of such histological changes.

This study has certain limitations. First, it was a single-center, retrospective study. If a high-quality multicenter or prospective cohort study is performed in the future, factors with a higher-level evidence affecting recurrence can be found. It seems that research in the long-term follow-up group after definitive surgery for PTs should be conducted. Second, we did not actually measure the patient's breast size. As a retrospective study, only mammography and ultrasonography remained, so it is impossible to measure the size of the breast. This should be supplemented in future studies.

This study is the first to present the correlation between the recurrence of PT and BMI, and a more extensive study should be conducted.

## Figures and Tables

**Table 1:** Clinicopathological characteristics of the study cohort.

Characteristic	All (*n* = 100)	Benign (*n* = 65)	Borderline (*n* = 29)	Malignant (*n* = 6)	*P* value
Median age: y (range)	44 (19–62)	44 (19–62)	45 (19–59)	40.5 (29–50)	*P* = 0.561
Median body Mass index: kg/m^2^ (range)	23.7 (16.1–31.9)	23.3 (16.1–31.9)	24.1 (17.0–31.4)	26.8 (21.5–31.8)	*P* = 0.219
Median pathological tumor size: cm (range)	4.0 (0.8–30.0)	3.5 (0.8–9.5)	6.5 (1.2–30.0)	6.5 (2.4–25.0)	*P* = 0.004

*Recurrence, n (%)*					*P* = 0.429
Not recurred	78 (78)	52 (80)	22 (76)	4 (67)	
Recurred	22 (22)	13 (20)	7 (24)	2 (33)	

*Surgery, n (%)*					*P* < 0.001
Wide excision	92 (92)	65 (100)	23 (79)	4 (67)	
Mastectomy	8 (8)	0 (0)	6 (21)	2 (33)	

*Stromal cellularity, n (%)*					*P* < 0.001
Mild	30 (30)	26 (40)	4 (14)	0 (0)	
Moderate	61 (61)	37 (57)	22 (76)	2 (33)	
Marked	7 (7)	0 (0)	3 (10)	4 (67)	
Unknown	2 (2)	2 (3)	0 (0)	0 (0)	

*Cellular atypia, n*					*P* < 0.001
Mild	70 (70)	61 (94)	9 (31)	0 (0)	
Moderate	21 (21)	2 (3)	19 (66)	0 (0)	
Marked	7 (7)	0	1 (3)	6 (100)	
Unknown	2 (2)	2 (3)	0 (0)	0 (0)	

*Mitosis, n*					*P* < 0.001
0–4	66 (66)	56 (86)	10 (35)	0 (0)	
5–9	15 (15)	4 (6)	11 (38)	0 (0)	
≤10	13 (13)	0	7 (24)	6 (100)	
Unknown	6 (6)	5 (8)	1 (3)	0 (0)	

*Borders, n*					*P* < 0.001
Pushing	14 (14)	1 (2)	9 (31)	4 (67)	
Infiltrative	84 (84)	62 (95)	20 (69)	2 (33)	
Unknown	2 (2)	2 (3)	0 (0)	0 (0)	

*Stromal overgrowth, n*					*P* < 0.001
No	79 (79)	61 (94)	15 (52)	3 (50)	
Yes	19 (19)	2 (3)	14 (48)	3 (50)	
Unknown	2 (2)	2 (3)	0 (0)	0 (0)	

*Heterologous component, n*					*P* = 0.008
No	95 (95)	62 (95)	29 (100)	4 (67)	
Yes	3 (3)	1 (2)	0 (0)	2 (33)	
Unknown	2 (2)	2 (3)	0 (0)	0 (0)	

*Final margin, n*					*P* = 0.065
Negative	68 (68)	47 (72)	19 (66)	2 (33)	
Positive	31 (31)	17 (26)	10 (34)	4 (67)	
Unknown	1 (1)	1 (2)	0 (0)	0 (0)	

**Table 2:** Factors associated with locoregional recurrence of phyllodes tumor.

Variables	No local recurrence	Local recurrence	HR (univariate)	95% CI (univariate)	*P* value	HR (multivariate)	95% CI (multivariate)	*P* value
*BMI*								
<23 kg/m^2^	29	14	Ref			Ref		
≥23 kg/m^2^	48	8	0.405	0.169–0.968	0.042	0.386	0.156–0.954	0.039

*OP name*								
Wide excision	71	21	Ref					
Mastectomy	7	1	0.753	0.101–5.635	0.782			

*Size*								
<5 cm	54	7	Ref			Ref		
≥5 cm	23	15	3.506	1.423–8.637	0.006	2.671	1.066–6.694	0.036

*Grade*								
Benign	52	13	Ref					
Borderline	22	7	0.872	0.346–2.197	0.772			
Malignant	4	2	1.327	0.298–5.920	0.711			

*Stromal hypercellularity*								
Mild	23	7	Ref					
Moderate	49	12	0.409	0.152–1.099	0.076			
Marked	4	3	1.089	0.277–4.284	0.903			

*Cellular atypia*								
Mild	56	14	Ref					
Moderate	15	6	1.005	0.384–2.626	0.992			
Marked	5	2	1.119	0.252–4.973	0.883			

*Mitosis*								
0–4	56	10	Ref					
5–9	8	7	2.821	1.060–7.505	0.038			
≤10	9	4	1.446	0.452–4.624	0.534			

*Tumor border*								
Pushing	11	3	Ref					
Infiltrative	65	19	1.232	0.361–4.211	0.739			

*Stromal overgrowth*								
No	65	14	Ref					
Yes	11	8	2.654	1.086–6.484	0.032	2.706	1.046–6.999	0.040

*Resection margin*								
Negative	57	11	Ref					
Positive	20	11	1.873	0.794–4.419	0.152			

HR, hazards ratio; CI, confidence interval; BMI, body mass index; OP, operation.

## Data Availability

The data used to support the findings of this study are available from the corresponding author upon request.

## References

[1] Choi N., Kim K., Shin K. H. (2019). The characteristics of local recurrence after breast-conserving surgery alone for malignant and borderline phyllodes tumors of the breast (KROG 16-08). *Clinical Breast Cancer*.

[2] Zhang Y., Kleer C. G. (2016). Phyllodes tumor of the breast: histopathologic features, differential diagnosis, and molecular/genetic updates. *Archives of Pathology & Laboratory Medicine*.

[3] Tan J., Ong C. K., Lim W. K. (2015). Genomic landscapes of breast fibroepithelial tumors. *Nature Genetics*.

[4] Tan P.-H., Jayabaskar T., Chuah K.-L. (2005). Phyllodes tumors of the breast. *American Journal of Clinical Pathology*.

[5] Lakhani S. E., Schnitt S. J. (2019). World health organization classification of tumours. *Breast Tumours*.

[6] Barrio A. V., Clark B. D., Goldberg J. I. (2007). Clinicopathologic features and long-term outcomes of 293 phyllodes tumors of the breast. *Annals of Surgical Oncology*.

[7] Laura H., Rogenberger S. M. T., Nimbkar S. N. (2020). Contemporary multi-institutional cohort of 550 cases of phyllodes tumors (2007-2017) demonstrates a need for more individualized margin guidelines. *Journal of Clinical Oncology*.

[8] Coltman C. E., Steele J. R., McGhee D. E. (2017). Breast volume is affected by body mass index but not age. *Ergonomics*.

[9] Chen W.-H., Cheng S.-P., Tzen C.-Y. (2005). Surgical treatment of phyllodes tumors of the breast: retrospective review of 172 cases. *Journal of Surgical Oncology*.

[10] Ben Hassouna J., Damak T., Gamoudi A. (2006). Phyllodes tumors of the breast: a case series of 106 patients. *The American Journal of Surgery*.

[11] Cheng S.-P., Chang Y.-C., Liu T.-P., Lee J.-J., Tzen C.-Y., Liu C.-L. (2006). Phyllodes tumor of the breast: the challenge persists. *World Journal of Surgery*.

[12] Belkacémi Y., Bousquet G., Marsiglia H. (2008). Phyllodes tumor of the breast. *International Journal of Radiation Oncology, Biology, Physics*.

[13] Spanheimer P. M., Murray M. P., Zabor E. C. (2019). Long-term outcomes after surgical treatment of malignant/borderline phyllodes tumors of the breast. *Annals of Surgical Oncology*.

[14] Tan P. H., Thike A. A., Tan W. J. (2012). Predicting clinical behaviour of breast phyllodes tumours: a nomogram based on histological criteria and surgical margins. *Journal of Clinical Pathology*.

[15] Spitaleri G., Toesca A., Botteri E. (2013). Breast phyllodes tumor: a review of literature and a single center retrospective series analysis. *Critical Reviews in Oncology*.

[16] Jang J. H., Choi M.-Y., Lee S. K. (2012). Clinicopathologic risk factors for the local recurrence of phyllodes tumors of the breast. *Annals of Surgical Oncology*.

[17] Co M., Chen C., Tsang J. Y., Tse G., Kwong A. (2018). Mammary phyllodes tumour: a 15-year multicentre clinical review. *Journal of Clinical Pathology*.

[18] Yom C. K., Han W., Kim S.-W., Park S. Y., Park I. A., Noh D.-Y. (2015). Reappraisal of conventional risk stratification for local recurrence based on clinical outcomes in 285 resected phyllodes tumors of the breast. *Annals of Surgical Oncology*.

[19] Kim S., Kim J.-Y., Kim D. H., Jung W. H., Koo J. S. (2013). Analysis of phyllodes tumor recurrence according to the histologic grade. *Breast Cancer Research and Treatment*.

[20] Lu Y., Chen Y., Zhu L. (2019). Local recurrence of benign, borderline, and malignant phyllodes tumors of the breast: a systematic review and meta-analysis. *Annals of Surgical Oncology*.

[21] Moo T.-A., Alabdulkareem H., Tam A. (2017). Association between recurrence and Re-excision for close and positive margins versus observation in patients with benign phyllodes tumors. *Annals of Surgical Oncology*.

[22] Sim Y., Tan V. K. M., Sidek N. A. B. (2018). Bilateral breast cancers in an Asian population, and a comparison between synchronous and metachronous tumours. *ANZ Journal of Surgery*.

